# Ramosetron for the treatment of irritable bowel syndrome with diarrhea: a systematic review and meta-analysis of randomized controlled trials

**DOI:** 10.1186/s12876-017-0734-2

**Published:** 2018-01-08

**Authors:** Qingqing Qi, Yan Zhang, Feixue Chen, Xiuli Zuo, Yanqing Li

**Affiliations:** Department of Gastroenterology, Qilu Hospital, Shandong University, 107 Wenhuaxi Road, Jinan, Shandong Province 250012 China

**Keywords:** Ramosetron, Irritable bowel syndrome with diarrhea, Efficacy, Safety, Meta-analysis

## Abstract

**Background:**

Ramosetron is a potent and selective serotonin type 3 receptor antagonist. This meta-analysis aimed to analyze the efficacy and safety of ramosetron for irritable bowel syndrome with diarrhea (IBS-D).

**Methods:**

Pubmed, MEDLINE, EMBASE and the Cochrane Library were searched for randomized controlled trials investigating the efficacy and safety of ramosetron for IBS-D. Risk of bias was assessed as described in the Cochrane handbook. A random effects model was used to calculate the effects of ramosetron vs placebo on symptomatic improvements, including relief of overall IBS symptoms, relief of abdominal discomfort/pain, improvement in abnormal bowel habits, and improvement in stool consistency, expressed as pooled relative risks (RRs) with 95% confidence interval (CI). Adverse events data were also summarized with RRs.

**Results:**

Four randomized controlled trials involving 1623 participants were included. Compared with placebo, ramosetron could lead to relief of overall IBS symptoms (RR 1.70; 95%CI 1.48, 1.95), relief of abdominal discomfort/pain (RR 1.41; 95%CI, 1.24, 1.59), improvement in abnormal bowel habits (RR 1.72; 95%CI, 1.50, 1.98) and improvement in stool consistency (RR 1.71; 95%CI 1.40, 2.08). Ramosetron could lead to relief of overall IBS symptoms in both male and female patients (RR; 95%CI: 1.94; 1.58, 2.38 and 1.49; 1.25, 1.79). The RR (95%CI) for reported adverse events of ramosetron vs placebo was 1.10 (0.97, 1.26) across all studies. No serious adverse events (e.g., ischemic colitis) were reported. The incidences of hard stool and constipation were higher in ramosetron group compared with placebo group (RR; 95%CI: 4.74; 3.00, 7.51 and 2.53; 1.57, 4.10, respectively).

**Conclusions:**

Ramosetron had beneficial effects to both male and female IBS-D patients. Treatment with ramosetron could cause more hard stool and constipation, without severe adverse events.

## Background

Irritable bowel syndrome (IBS) is characterized as abdominal pain and/or discomfort with altered bowel habits, without any demonstrable structural and biochemical abnormalities [1]. Based on the predominant bowel habit, Rome III criteria categorize IBS into four subtypes: IBS with diarrhea (IBS-D), IBS with constipation (IBS-C), mixed IBS (IBS-M), and unsubtyped IBS. IBS affects an estimated 10-15% of adults worldwide [2–5]. IBS leads to significant reduction in patients’ daily activities and quality of life [3, 6, 7], and accounts for a considerable amount of medical resources [8–10]. Therefore, development of effective, well-tolerated and safe IBS treatments is important for the patients, health-care systems, and society as a whole.

Serotonin type 3 (5-HT_3_) receptor is one of the seven subtypes serotonin (5-HT) receptors. This receptor has been identified on the intestinal nerves, and a number of studies have suggested its role in the pathogenesis of IBS [11, 12]. Treatment targeted at 5-HT_3_ receptor has been the focus of numbers of recent studies. Clinical trials and meta-analysis have confirmed the efficacy of 5-HT_3_ receptor antagonist alosetron in the treatment of IBS-D patients [13–17]. However, some serious gastrointestinal events (e.g., ischemic colitis and severe constipation) have been reported with alosetron use [14, 18]. Therefore, alosetron is only available exclusively for women with severe, chronic IBS-D refractory to conventional therapy in the USA.

Ramosetron is a potent and selective 5-HT_3_ receptor antagonist. It has been used as an antiemetic for cancer patients in their chemotherapy and for the prevention of postoperative nausea and vomiting for many years [19–21]. Clinical trials have evaluated the efficacy and safety of ramosetron in IBS-D patients [22–26]. Though the results of these available clinical trials have been generally favorable, there is still no evidence for the efficacy of ramosetron for IBS-D based on the systematic review and meta-analysis. The efficacy of ramosetron on the treatment of both male and female IBS-D population, and the safety data of ramosetron, need to be clearly elucidated. Therefore, we performed this systematic review and meta-analysis of randomized, controlled trials (RCTs) comparing ramosetron with placebo in the treatment of IBS-D, in order to obtain more accurate and comprehensive results to systematically evaluate the efficacy and safety of ramosetron in the treatment of abdominal symptoms and bowel function in IBS-D.

## Methods

This meta-analysis was conducted according to the Cochrane collaboration’s systematic review Methodology [27] and reported according to the Preferred Reporting Items for Systematic Reviews and Meta-Analyses (PRISMA) [28].

### Search strategy

Pubmed, MEDLINE, EMBASE and the Cochrane Library were systematically searched from inception to November 2015. The following search terms were used: “IBS”, “irritable bowel syndrome” AND “ramosetron”, “ramosetron hydrochloride”. No language or document type restrictions were applied. All bibliographies of the identified relevant studies were also checked to identify any additional studies. After scanning titles and abstracts of articles selected from the initial search, we read through the full text of eligible articles. Two reviewers independently performed the search process and disagreements were resolved by discussion.

### Study selection

Studies meeting the following criteria were included: adults patients (age above 18 years) with IBS-D diagnosed by Rome criteria; randomized, double-blind, placebo-controlled trials that compared the efficacy of ramosetron vs placebo; included dichotomous assessment of response to therapy in term of effect on global IBS symptoms, abdominal pain/discomfort or abnormal bowel habits. The exclusion criteria were as follows: no placebo group; review article; incomplete data; abstract later published for full-text article.

### Outcome assessment

The primary measurements were the effects of ramosetron compared with placebo on symptomatic improvements, including relief of overall IBS symptoms, relief of abdominal discomfort/pain, improvement in abnormal bowel habits, and improvement in stool consistency. The secondary measurement was the adverse events occurring as a result of therapy, aiming to evaluate the safety of ramosetron.

### Data extraction

All the data of the primary and secondary measurements were extracted as dichotomous outcomes based on criteria of treatment response defined by the authors of included studies. In addition, the following clinical data were extracted for each trial: country of origin, criteria used to define IBS, number of patients in each group, ramosetron dose, treatment duration, follow-up duration after therapy, and outcomes. Data were extracted as intention-to-treat analyses, with all dropouts assumed to be treatment failures, wherever trial reporting allowed this. All data were extracted independently by two investigators on to a Microsoft Excel spreadsheet. Disagreements were resolved after discussion with other investigators.

### Risk of bias assessment

According to the Cochrane Handbook for Systematic Reviews of Interventions, risk of bias assessment tool could be used for study quality assessment of randomized control trials. Risk of bias was assessed as described in the Cochrane handbook [27]. This evaluates the random sequence generation, allocation concealment, whether blinding was implemented, the completeness of follow-up, whether there was evidence of selective reporting of outcomes, and other biases. Two review authors independently judged the risk of bias and disagreements were resolved by discussion.

### Data synthesis and statistical analysis

All statistical analysis were performed using the Cochrane Collaboration software Review Manager (version 5.3, The Nordic Cochrane Centre, Copenhagen, Denmark). The effect of ramosetron in IBS was expressed as a relative risk (RR) with 95% confidence interval (CI) of symptomatic improvement with ramosetron compared with placebo, based on intention-to-treat analysis. Adverse events data were also summarized with RRs. The heterogeneity across the included studies was assessed using both the I^2^ statistic with a cutoff of ≥50% and the χ2-test with a *P* value <0.10. The pooled RRs with 95%CIs were calculated using a random effects model, in order to provide a more conservative estimate of the effect of ramosetron. The sensitivity analysis was performed by excluding each of the studies at a time and re-analyze the remaining studies. Given the small number of studies included in our analysis, we did not performed the statistical tests for assessing publication bias [29].

## Results

### Descriptions of included studies

**Fig. 1:**
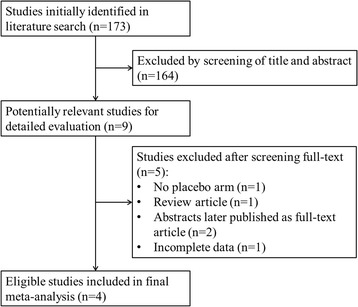
Flow chart of literature search and selection

Figure 1 shows the flow chart of study selection process. A total of 173 studies were identified in initial literature search. Based on the inclusion and exclusion criteria, 164 studies were excluded after screening the titles and abstracts. The full texts of the remaining 9 studies were thoroughly reviewed, and another 5 studies were excluded due to the following reasons: no placebo arm (*n* = 1), review article (*n* = 1), abstracts later published as full-text articles (*n* = 2) and incomplete data (*n* = 1). Finally, four RCTs of ramosetron vs placebo in IBS-D were included in the final meta-analysis [22, 23, 25, 26]. In this section of our study the coefficient of agreement between the two authors performing the study search was 1.0, which meant that there was no disagreement between the two authors. The reason for this was that both authors strictly followed the search criteria we established in this section. They used the same search terms in the same databases with no language or document type restrictions. All bibliographies of the identified relevant studies were also checked carefully to identify any additional studies, and both authors read through the full text of eligible articles after scanning titles and abstracts of articles selected from the initial search.

In the four RCTs, a total of 1623 participants (812 in ramosetron group and 811 in placebo group) were included. The clinical characteristics of included studies are listed in Table 1. All four studies were conducted in Japan. Two studies defined IBS-D based on Rome II criteria [25, 26], and another two studies used Rome III criteria [22, 23]. The treatment duration in all studies were 12 weeks, and the follow-up duration were also 12 weeks. All included studies used the same definition for the improvement of IBS symptoms.

**Table 1:** Characteristics of randomized controlled trials of ramosetron vs placebo in irritable bowel syndrome with diarrhea

Study	Country	Criteria for IBS	Sample size (ramosetron vs placebo)	Dose	Treatment duration	Follow-up duration	Primary outcomes	Secondary outcomes
Matsueda K 2008a [25]	Japan	Rome II	270 vs 269	5 μg	12 weeks	12 weeks	Relief of overall IBS symptoms	1.Relief of abdominal discomfort/pain 2.Improvement in abnormal bowel habits 3.Assessment of IBS symptoms^a^
Matsueda K 2008b [26]	Japan	Rome II	103 vs 109	5 μg	12 weeks	12 weeks	Relief of overall IBS symptoms	1.Relief of abdominal discomfort/pain 2.Improvement in abnormal bowel habits 3.Assessment of IBS symptoms^a^
Fukudo S 2014 [22]	Japan	Rome III	147 vs 149	5 μg	12 weeks	12 weeks	Improvement in stool consistency	1.Relief of overall IBS symptoms 2.Relief of abdominal discomfort/pain 3.Improvement in abnormal bowel habits 4.Assessment of IBS symptoms^a^ 5.IBS-QOL
Fukudo S 2015 [23]	Japan	Rome III	292 vs 284	2.5 μg	12 weeks	12 weeks	1.Relief of overall IBS symptoms 2.Improvement in stool consistency	1.Relief of abdominal discomfort/pain 2.Improvement in abnormal bowel habits 3.Assessment of IBS symptoms^a^ 4.IBS-QOL

*IBS-QOL*, irritable bowel syndrome-quality of life

^a^Assessment of IBS symptoms included severity of abdominal discomfort and/or pain, stool form, stool frequency, urgency and feeling of incomplete bowel movement

**Fig. 2:**
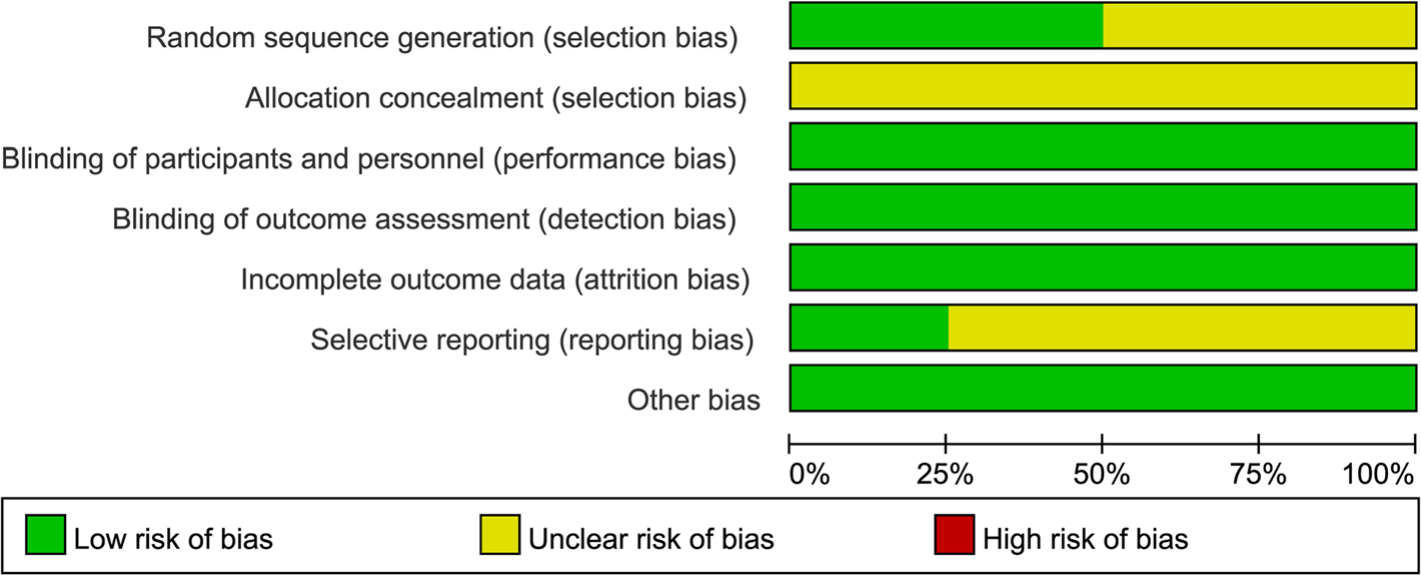
Risk of bias assessment for included studies

Figure 2 shows the risk of bias for all studies assessed using the Cochrane Collaboration tool. Two studies did not describe the detail of sequence generation process, leading to unclear risk of bias in the domain of “random sequence generation”. All four studies did not describe the method of concealment in sufficient detail, leading to unclear risk of bias in the domain of “allocation concealment”. All studies reported the mainly expected primary and secondary outcomes. However, in three studies some detailed changes of symptoms were not reported. We defined these studies as unclear risk of bias in the domain “selective reporting”. Low risk of bias was seen for all studies in the domains of “blinding of participants and personnel”, “blinding of outcome” and “incomplete outcome data”. There was no other bias.

### Efficacy of Ramosetron in the treatment of IBS-D

#### Effect on relief of overall IBS symptoms

**Fig. 3:**
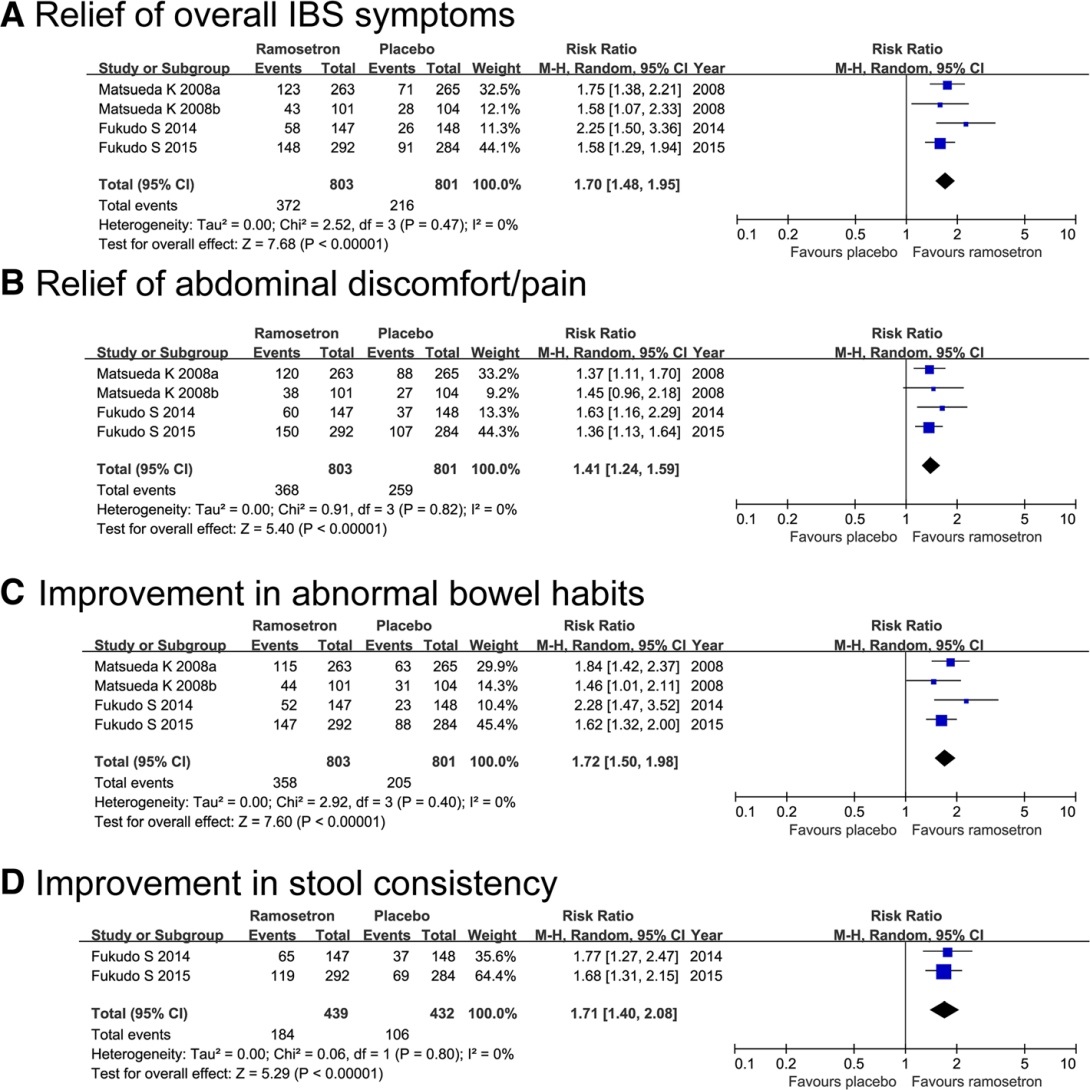
Forest plot of ramosetron vs placebo in irritable bowel syndrome with diarrhea. Effect on relief of overall IBS symptoms (**a**), relief of abdominal discomfort/pain (**b**), improvement in abnormal bowel habits (**c**), and improvement in stool consistency (**d**)

All four included studies evaluated the effect of ramosetron on relief of overall IBS symptoms. A total of 812 patients were assigned to the ramosetron group, whereas 811 patients were assigned to the placebo group. As shown in Fig. 3a, the RR of relief of overall IBS symptoms after treatment with ramosetron vs placebo was 1.70 (95%CI 1.48, 1.95). No heterogeneity was detected across all studies (*P* = 0.47, I^2^ = 0%).

**Fig. 4:**
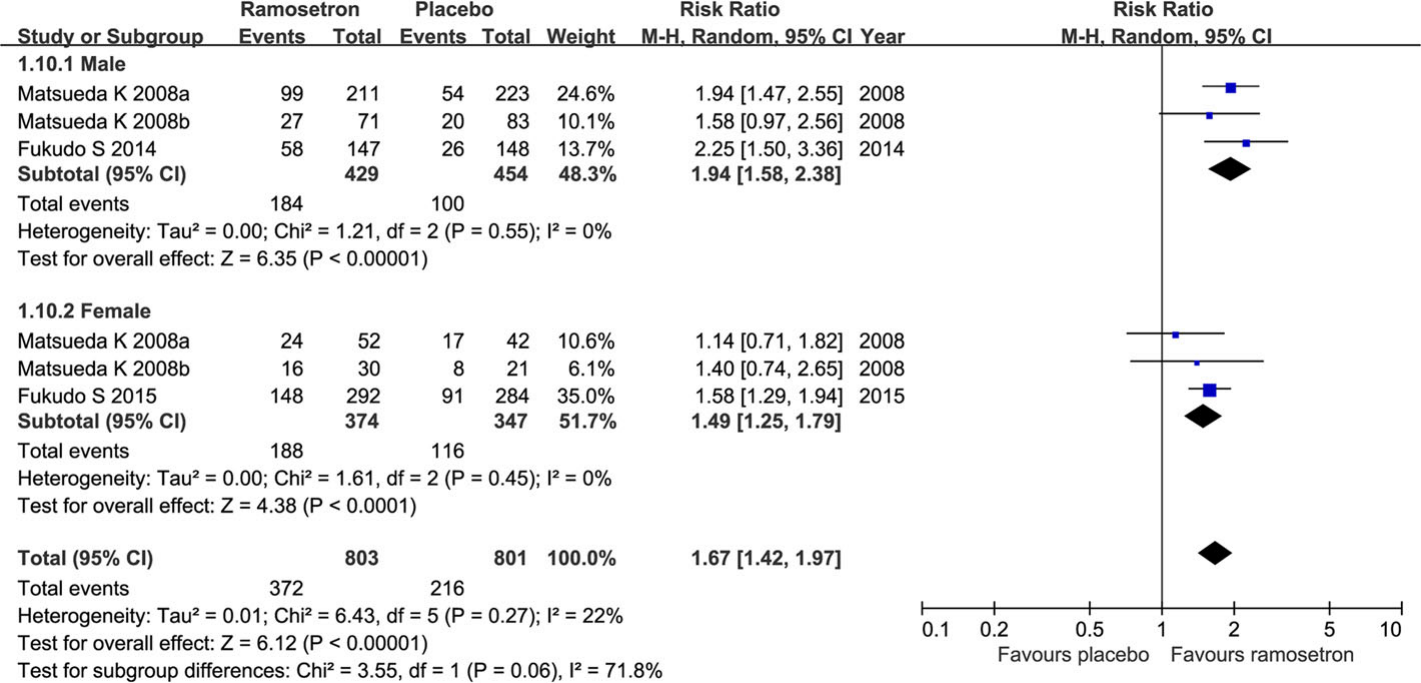
Forest plot of ramosetron vs placebo in irritable bowel syndrome with diarrhea. Effect on relief of overall IBS symptoms in male and female groups

For the outcome of relief of overall IBS symptoms, the RR was 1.94 (95%CI 1.58, 2.38) for male, and 1.49 (95%CI 1.25, 1.79) for female, indicating that treatment with ramosetron could lead to significant relief of overall IBS symptoms for both male and female patients. (Fig. 4).

#### Effect on relief of abdominal discomfort/pain

All studies assessed the relief of abdominal discomfort/pain. Ramosetron resulted in a pooled RR of response of 1.41 (95% CI, 1.24, 1.59) compared with placebo. There was no heterogeneity across all studies (*P* = 0.82, I^2^ = 0%). (Fig. 3b).

#### Effect on improvement in abnormal bowel habits

All four studies evaluated the effect of ramosetron on improvement in abnormal bowel habits. The pooled RR (95% CI) for improvement in abnormal bowel habits was 1.72 (1.50, 1.98). There was no heterogeneity across all studies (*P* = 0.40, I^2^ = 0%). (Fig. 3c).

#### Effect on improvement in stool consistency

Two of the four studies reported the outcome of improvement in stool consistency [22, 23]. Ramosetron showed significant improvement in stool consistency compared with placebo (RR 1.71; 95% CI 1.40, 2.08). No heterogeneity was observed (*P* = 0.80, I^2^ = 0.0%). (Fig. 3d).

### Sensitivity analysis

Due to the difference in the sample size of the component RCTs, a sensitivity analysis was performed by excluding each of the studies at a time and re-analyze the remaining. The results of sensitivity analysis in this study are shown in Table 2. Although the relative risk changed, the overall conclusions were still the same, which meant that none of the studies had an undue influence on the final relative risk.

**Table 2:** Sensitivity analysis of the effects of ramosetron vs placebo in irritable bowel syndrome with diarrhea

	Study removed	Ramosetron		Placebo		Pooled relative risks(RRs) with 95% confidence interval(CI)
		Events	Total	Events	Total	
Relief of overall IBS symptoms	None	372	803	216	801	1.70 [1.48, 1.95]
	Matsueda K 2008a [25]	249	540	145	536	1.70 [1.40, 2.06]
	Matsueda K 2008b [26]	329	702	188	697	1.73 [1.47, 2.03]
	Fukudo S 2014 [22]	314	656	190	653	1.64 [1.42, 1.89]
	Fukudo S 2015 [23]	224	511	125	517	1.80 [1.50, 2.15]
Relief of abdominal discomfort/pain	None	368	803	259	801	1.41 [1.24, 1.59]
	Matsueda K 2008a [25]	248	540	171	536	1.43 [1.22, 1.66]
	Matsueda K 2008b [26]	330	702	232	697	1.40 [1.23, 1.60]
	Fukudo S 2014 [22]	308	656	222	653	1.38 [1.20, 1.57]
	Fukudo S 2015 [23]	218	511	152	517	1.44 [1.22, 1.71]
Improvement in abnormal bowel habits	None	358	803	205	801	1.72 [1.50, 1.98]
	Matsueda K 2008a [25]	243	540	142	536	1.69 [1.38, 2.06]
	Matsueda K 2008b [26]	314	702	174	697	1.77 [1.52, 2.06]
	Fukudo S 2014 [22]	306	656	182	653	1.66 [1.44, 1.93]
	Fukudo S 2015 [23]	211	511	117	517	1.80 [1.46, 2.23]
Improvement in stool consistency	None	184	439	106	432	1.71 [1.40, 2.08]
	Fukudo S 2014 [22]	119	292	69	284	1.68 [1.31, 2.15]
	Fukudo S 2015 [23]	65	147	37	148	1.77 [1.27, 2.47]

### Safety

**Fig. 5:**
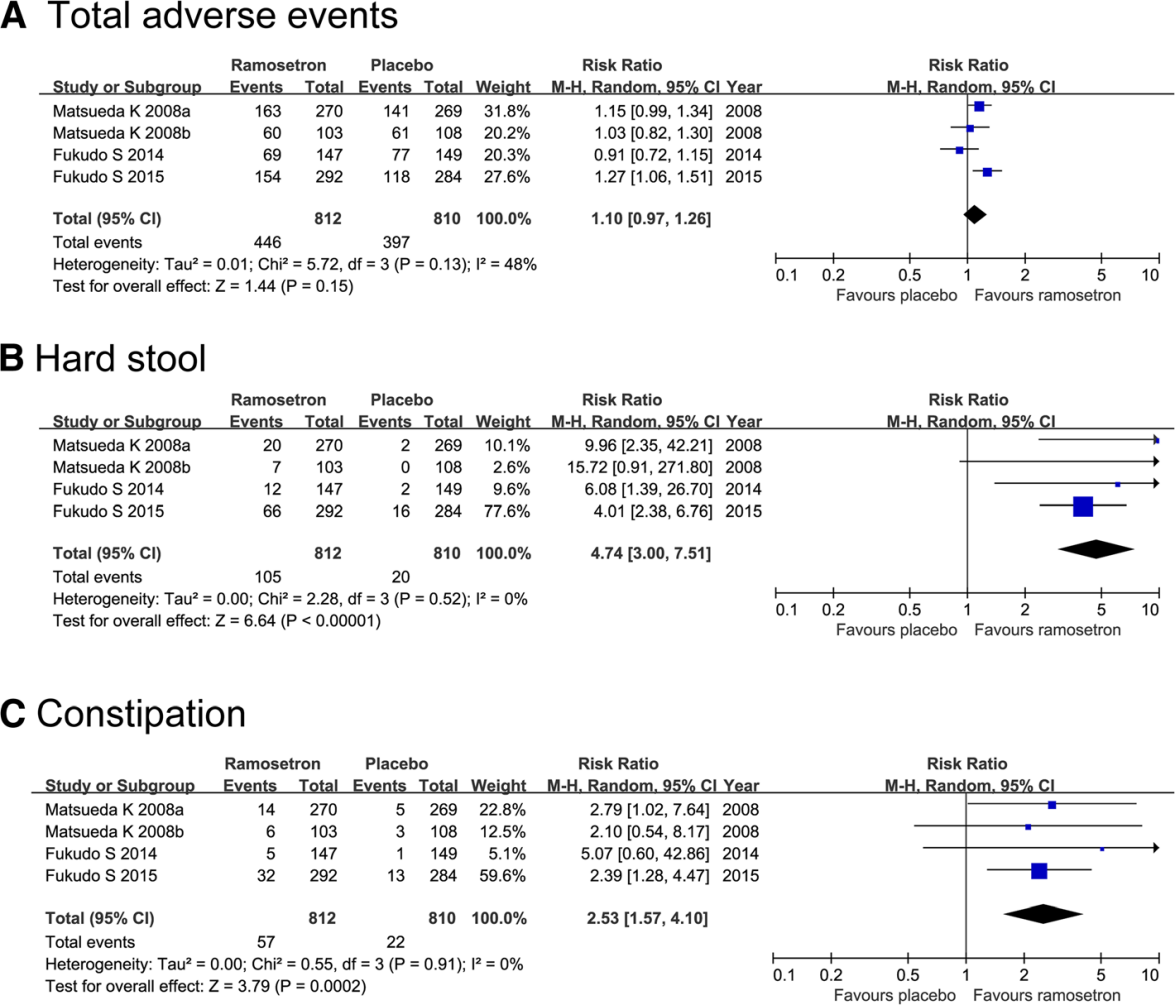
Forest plot of ramosetron vs placebo in irritable bowel syndrome with diarrhea on safety evaluation. **a** Total adverse events. **b** Hard stool. **c** Constipation

As shown in Fig. 5, safety profile was evaluated in all the included studies. In total, 54.9% of patients assigned to ramosetron experienced adverse events, compared with 49.0% of patients allocated to placebo. The RR (95%CI) for reported adverse events of ramosetron in comparison with placebo was 1.10 (0.97, 1.26) across all studies. There were no serious adverse events, especially no cases of ischemic colitis and severe constipation were reported. The most common adverse events with usage of ramosetron were hard stool and constipation. When data were pooled, the incidence of hard stool and constipation were both significantly higher in patients randomized to ramosetron than those receiving placebo, as the RRs (95% CI) were 4.74 (3.00, 7.51) and 2.53 (1.57, 4.10), respectively.

## Discussion

This systematic review and meta-analysis confirmed the benefits of ramosetron over placebo in patients with IBS-D. Treatment with ramosetron was associated with symptomatic improvements, including relief of overall IBS symptoms, relief of abdominal discomfort/pain, improvement in abnormal bowel habits, and improvement in stool consistency. Treatment with ramosetron showed efficacy on both male and female patients. Hard stool and constipation occurred more frequently in patients on ramosetron compared with placebo. However, ramosetron is considered to be safe and well tolerated, because no severe adverse events were reported and the incidence of total adverse events was similar in ramosetron and placebo arm. Its clear efficacy and low incidence of serious adverse events suggest that ramosetron is a promising therapeutic agent for IBS-D patients.

Although great progress has been achieved in the understanding of IBS, its exact mechanism remains obscure and currently conventional treatments remain unsatisfied. As summarized by American College of Gastroenterology, the quality of evidence for a lot of currently available managements for IBS, such as dietary manipulation, certain antispasmodics, peppermint oil, non-absorbable antibiotics, etc. were graded as “very low”, “low” or “moderate”. Therefore, we still lack sufficiently effective treatments for IBS [30]. It is certainly of significance to provide strong evidence for the novel drugs used for IBS. Although the results of previously trials have been generally favorable, our present study provides an evidence of systematic review and meta-analysis for the efficacy and safety of ramosetron for IBS-D. What is more, the similarity across all eligible trials, including enrollment criteria (Rome criteria), design (randomized, double-blind, placebo-controlled), outcome measurements (same outcomes and same definition of outcomes), and treatment and follow-up duration (12 weeks), results in high comparability of our results and makes the pooled estimates robust and convincing.

In consideration of the involvement of 5-HT and the 5-HT_3_ receptors in the pathogenesis of IBS, a number of clinical trials have been conducted to evaluate the efficacy of 5-HT_3_ antagonists. Most of these trials were of good quality, but it was noteworthy that these kinds of drugs were associated with a potential risk of ischemic colitis and cardiovascular events respectively [18]. These risks largely limit the widely clinical application of these drugs. For instance, alosetron is only available exclusively for women with severe, chronic IBS-D refractory to conventional therapy in the USA. In a recently published open-label, uncontrolled, long-term safety trial, constipation occurred in 19.7% of IBS-D patients receiving 2.5 μg and 10.5% of patients receiving 5 μg of ramosetron, and there was no serious adverse event related to ramosetron, especially ischemic colitis [31]. In our meta-analysis, there was no significant difference in the incidence of total adverse events in ramosetron group and placebo group (RR 1.10; 95%CI 0.97, 1.26). Ramosetron did lead to more hard stool and constipation, which are considered to be the classic effect of 5-HT_3_ receptor antagonist. However, there were no severe adverse events, especially no case of ischemic colitis were reported. In addition, the incidence of constipation due to administration of ramosetron was only 7.02%, which was much lower than that in alosetron group (23.9%) reported previously [14]. This is extremely important due to the possible relationship between the ischemic colitis and constipation [32]. Lower incidence of constipation in ramosetron than in alosetron means ramosetron is less likely cause ischemic colitis. All these data demonstrate that ramosetron is not only effective, but also safe for the treatment of IBS-D patients.

The 5-HT_3_ receptor antagonist alosetron has been approved for only female patients with IBS-D, since the efficacy of alosetron has not been confirmed in males [33]. Development of effective drugs for male IBS-D patients is therefore in urgent need. The efficacy of ramosetron has been evaluated in both male and female populations in previous trials [22, 23, 25, 26]. In our meta-analysis, we pooled the dichotomous data of relief of overall IBS symptom. For both male and female patients, ramosetron could lead to significant relief of overall IBS symptoms (RR 1.94; 95%CI 1.58, 2.38 vs RR 1.49; 95%CI 1.25, 1.79. respectively). These pooled data revealed that ramosetron should be recommended for both male and female IBS-D patient in clinical practice. Many possible factors, including sexual cycle, biobehavioral responses to stress, differences in roles and emotions between males and females, may affect gender differences in the response to treatments with alosetron and ramosetron [34]. Furthermore, alosetron could transfer to the brain while ramosetron acts only on peripheral tissues, which may also contribute to the gender differences in response to different 5-HT_3_ receptor antagonists [26]. The exact mechanism of gender differences in the treatment response need to be further investigated.

Although most outcomes in the included studies are subjective and were measured on an ordinal scale, and were analyzed as dichotomous outcomes in the meta-analysis, the definitions for symptom relief were consistent across studies. In all included studies, the monthly responder was defined as a patient who had experienced “Completely relieved” or “Considerably relieved” for at least 2 weeks of the 4-week treatment, and the monthly responder rate of “symptom relief in IBS” was analyzed in these studies. What is more, in these different studies subjective outcomes were measured using standardized and validated tools. Severity of abdominal pain and discomfort was assessed daily on a 5-point scale (0: none, 1: mild, 2: moderate, 3: severe, 4: intolerable), and stool form data was scored on a 7-point ordinal scale according to the Bristol Stool Form Scale. Above consistent definitions for symptom relief and standardized and validated tools used for subjective outcomes measurement all make the pooled estimates in our meta-analysis robust and convincing.

There are several limitations in our present meta-analysis. First, the absolute number of clinical trials included is small. Although this might be partly compensated by the relative large amount of participants enrolled in each trial, further clinical trials are necessary to establish a more powerful evidence for the use of ramosetron in IBS-D. We did not evaluate the publication bias, also due to insufficient (less than 10) eligible studies included in our analysis, in line with recent recommendations [29]. Second, all of the included trials are conducted in Japan. This could limit the generalizability of the results to broader populations worldwide. The efficacy and safety of another 5-HT_3_ receptor antagonist alosetron have been demonstrated in Western populations [13, 15]. The efficacy and safety data of ramosetron for the treatment of IBS-D in Western population are essential and in urgent need. Third, all the included trials had high quality, however, the blinding methods used in these trials are not sufficiently described, especially for the allocation concealment. Further better designed randomized, placebo-controlled trials optimized to reduce bias are needed to reveal the efficacy of ramosetron in a broader population worldwide.

## Conclusions

In conclusion, the significant pooled effect estimates in symptomatic improvements in ramosetron compared with placebo, combined with a good safety and tolerability profile, suggest that ramosetron is a significant development in the treatment of IBS-D. Treatment with ramosetron has similar efficacy for male and female patients. The efficacy of the drug in a broader population, especially in Western population, deserves further investigation in better designed clinical trials.

## Abbreviations

5-HT: Serotonin
5-HT_3_: Serotonin type 3
CI: Confidence interval
IBS: Irritable bowel syndrome
IBS-C: Irritable bowel syndrome with constipation
IBS-D: Irritable bowel syndrome with diarrhea
IBS-M: Mixed irritable bowel syndrome
RCTs: Randomized, controlled trials
RR: Relative risk
